# Relationship Between Price and Diagnosis-Related Group Tariff for Medical Devices Assessed by a Regional Health Technology Assessment Committee

**DOI:** 10.7759/cureus.23092

**Published:** 2022-03-12

**Authors:** Sabrina Trippoli, Andrea Messori, Giovanna Borselli, Filomena Autieri, Domenica Mamone, Claudio Marinai

**Affiliations:** 1 Health Technology Assessment (HTA) Unit, Regione Toscana, Firenze, ITA; 2 Hospital Pharmacy, Usl Toscana Centro, Florence, ITA; 3 Direzione Sanitaria, Azienda Ospedaliero Universitaria Careggi, Florence, ITA; 4 Hospital Pharmacy, Azienda Ospedaliero Universitaria Pisana, Pisa, ITA; 5 Drugs and Medical Devices, Tuscany Region, Florence, ITA

**Keywords:** hta, value-based purchasing, drg-s, cost-effectiveness analysis, high-cost medical devices

## Abstract

Introduction

Medical devices (MDs) make up an important share of total in-hospital expenditure. At the level of individual patients, this share is represented by the ratio of the cost of MD incurred by the patient vs. the total cost of in-hospital care for the same patient. If tariffs rather than costs are considered, the denominator of this ratio is given by the diagnosis-related group (DRG) and the ratio is the cost of MD over DRG tariff. The objective of this paper is to present a retrospective analysis comparing the ratio of price vs. DRG tariff for a group of devices belonging to risk class III or active implantable. These devices are those assessed in the years 2020 and 2021 by two committees of the Tuscany region in Italy.

Materials and methods

The information on price and DRG was taken from the health technology assessment (HTA) reports concerning MDs evaluated by the two above-mentioned regional committees in the years 2020 and 2021. In these reports, the information on the cost-effectiveness ratio was reported for a subset of MDs. In all cases, a preliminary qualitative assessment was carried out to determine the presence or absence of a healthcare impact in the post-discharge phase. In these preliminary analyses, the perspective of NHS was adopted.

Results

Our analysis was focused on 24 devices of either class III or active implantable. According to our results, a wide variability was found in the ratios between device price and DRG associated with its use. This ratio ranged from a minimum of about 3% in the case of the Hyalobarrier gel (Nordic Pharma GmbH, Zürich, Switzerland) for post-surgical adhesion to a maximum of 132% in the case of the Neovasc Reducer (EPS Vascular AB, Viken, Sweden), a device indicated in the narrowed coronary sinus. Three devices, i.e., PuraStat (3-D Matrix, Ltd., Tokyo, Japan), Ascyrus Medical Dissection Stent (AMDS, CryoLife, Inc., Kennesaw, GA), and Tendyne (Abbott Cardiovascular, Plymouth, MN), were found to be priced more than the reimbursement tariff (i.e., ratio > 100%). Ratios between 50% and 100% were found in about half of the devices. From our preliminary assessment on the presence of a post-discharge impact, 15 devices out of 24 (62%) were found to determine a substantial impact, while the remaining nine (38%) did not. In general, when costs and benefits of a device do not extend beyond the patients’ discharge, the presence of a ratio > 100% reliably suggests the conclusion that the device price needs to be reduced and/or the tariff needs to be increased. On the other hand, in cases where the device extends its impact beyond the patient’s hospital stay, the decision of reducing price or increasing tariff becomes more complex, and so these adjustments cannot be determined unless more information on some critical aspects is made available.

Conclusions

Until the above-mentioned improvements do not take place, rational interventions on DRG are virtually unfeasible owing to this lack of critical information. On the other hand, it is also difficult to intervene on device prices, again owing to the lack of critical information.

## Introduction

Some classes of medical devices (MDs) make up an important share of total in-hospital expenditure [1]. When calculated for a given hospital, this share of expenditure for MDs over total healthcare expenditure plays an important role in the governance of MDs. At the level of individual patients, the same index is represented by the device cost divided by the total cost of in-hospital care for the same patient. If diagnosis-related group (DRG) tariffs rather than costs are considered, the denominator of this ratio is given by the DRG and the ratio is device cost over DRG tariff. It is well known that the values of this ratio may vary widely across different procedures. The international literature on this topic is very fragmented in that most articles published in the past years refer to a single device or procedure for which cost, benefits, and tariffs were estimated. Well-conducted reviews on this topic involving multiple devices are lacking. The PubMed search described in the Appendix clearly confirms this conclusion.

As regards expenditures and tariffs related to MDs, a wide variability occurs between different regions on a national basis and also between different hospitals within the same region. For some interventions or procedures that involve the use of very expensive MD, the above ratio may exceed the limit of 100%, and in some cases to a large extent. As a partial solution to this critical issue, some regions have adopted local increases for a subset of DRG tariffs [2], which are generally applied to cases where the ratio is much higher than 100%. This solution is clearly imperfect because it fails to offer a systematic countermeasure to the problem.

Hence, the governance of in-hospital expenditure for MDs is complex [1]. To evaluate the economic implications of the use of certain MDs, sophisticated analyses within the National Health Service (NHS) would be needed so that the cost-effectiveness of the device can be estimated. Cost-effectiveness should represent the main driver influencing the procurement of these products. In particular, if cost-effectiveness is systematically determined for all products belonging to the same class, a comparison can be made between a new MD proposed for in-hospital use and those already available. Furthermore, another relevant issue is the impact in clinical and economic terms that the device may determine after a patient's discharge. When this post-discharge impact is substantial, the analysis becomes more complex; when this impact is negligible, the analysis is more straightforward.

The objective of this paper is to present a retrospective analysis comparing the ratio of MD price versus DRG tariff for a group of devices belonging to risk class III or active implantable. These devices were those assessed in the years 2020 and 2021 by the two committees responsible for these decisions in the Tuscany region, namely, the group of regional professionals responsible for MD evaluations (Gruppo Regionale Dispositivi Medici (GRDM)) and the regional committee for health technology assessment (Commissione Health Technology Assessment (CHTA)). The present report also includes the information, when available, on the cost-effectiveness of these devices. Finally, a qualitative assessment is reported for each device about the presence or absence of a post-discharge clinical and/or economic impact.

## Materials and methods

The information on price, DRG, and any further parameter concerning cost-effectiveness was taken from the HTA reports of MDs evaluated by the two above-mentioned regional committees in the years 2020 and 2021. These reports are published in the specific section of the website of the Tuscany region [3]. In these reports, the information on cost-effectiveness, when available, is of fundamental importance because it allows comparing the clinical benefit with the cost of the MD concerned and, in general, to compare the cost-effectiveness profiles across devices belonging to the same therapeutic class. In cases where a formal study on the cost-effectiveness ratio was not available for the MD concerned, a preliminary qualitative assessment was carried out to determine the presence or absence of a healthcare impact (clinical and/or economic) in a post-discharge time horizon. In these preliminary analyses, this impact was assessed according to the NHS perspective, which includes both the hospitalization period and the post-discharge period. The societal perspective was not adopted because this would have required the evaluation of indirect costs as well. In the first place, these analyses allowed to identify the situations where this post-discharge impact does not occur; in such cases, a very simple analysis appears to be acceptable in which the analytical perspective is restricted to the hospital (without extending to the community) and in which factors determining cost and effectiveness result from patient's hospital stay. On the other hand, the more complex situations are those where this post-discharge impact occurs and is relevant in clinical and economic terms. In these cases, the economic analysis is complex because the NHS perspective must be applied so that determinants of cost and effectiveness can be thoroughly assessed after the patient’s discharge.

## Results

**Figure 1:**
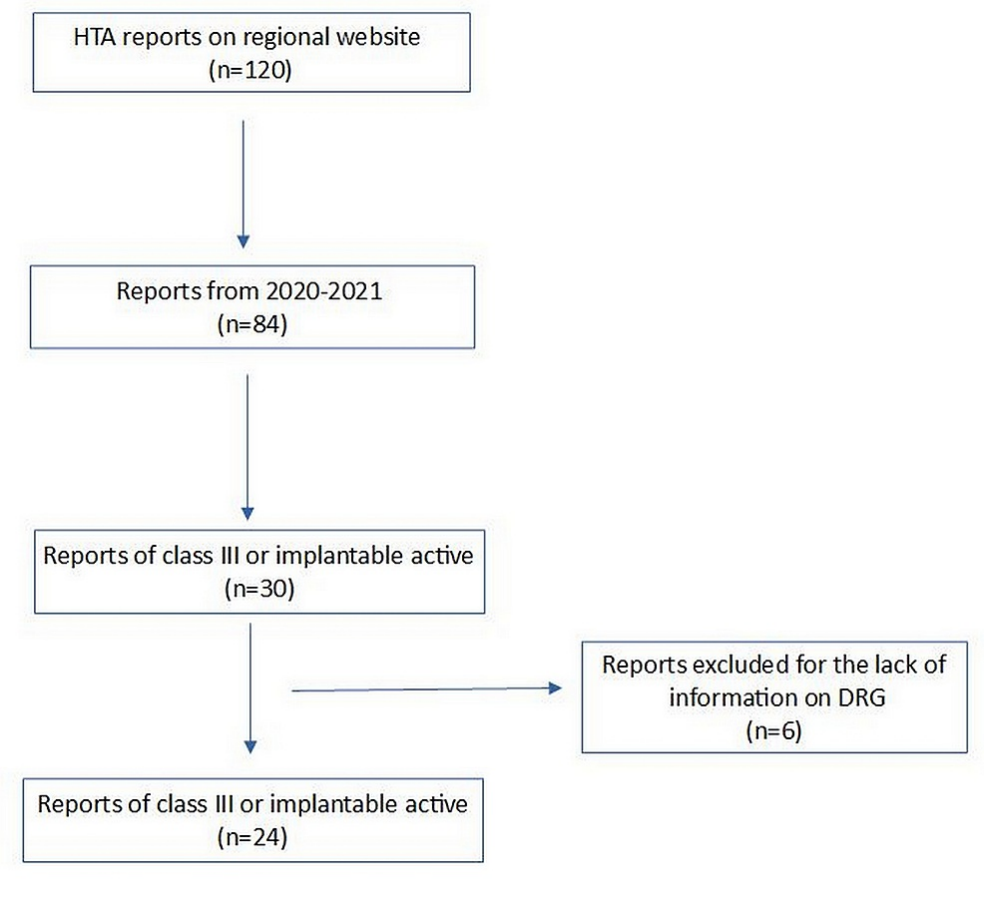
Flow diagram describing our research. HTA, health technology assessment; DRG, diagnosis-related group.

The analysis included 24 (80%) of the 30 devices of class III or active implantable evaluated in 2020 and 2021 by GRDM and CHTA. The six devices left out from the present report were excluded owing to the lack of basic information needed for our analysis. Figure 1 shows the flow diagram of our analysis.

In our results (Table 1), a wide variability was found in the ratio between device price and DRG associated with its use. This ratio ranged from a minimum of about 3% in the case of the Hyalobarrier gel (Nordic Pharma GmbH, Zürich, Switzerland) for post-surgical adhesion to a maximum of 132% in the case of the Neovasc Reducer (EPS Vascular AB, Viken, Sweden), a device indicated in the narrowed coronary sinus.

**Table 1:** Ratio of MD price versus DRG tariff, post-discharge impact, and cost-effectiveness ratio of MD. * Diagnosis-related group; § regional tariff, see [2]; ** clinical and/or economic impact. MD, medical device; DRG, diagnosis-related group.

Name of the device	Unit price (euro)	DRG code^*^ (description)	DRG tariff^§^ (euro)	Ratio of MD price versus DRG tariff (%)	Post-discharge impact determined by the MD^**^ (Y/N)	Cost-effectiveness analysis of MD
1. Neovasc Reducer (EPS Vascular AB, Viken, Sweden): coronary sinus reducer stent	6,500	556 (Percutaneous interventions on the cardiovascular system without major cardiovascular diagnosis with bare-metal stents)	4,889	132.0	Yes	The cost-effectiveness ratio is favorable considering a Neovasc Reducer price of 7,000 euros [4]
2. PuraStat (3-D Matrix, Ltd., Tokyo, Japan): hemostatic hydrogel	300	412 (Anamnesis of malignant neoplasm with endoscopy)	230	130.4	No	Not available
3. Ascyrus Medical Dissection Stent, AMDS (CryoLife, Inc., Kennesaw, GA): hybrid aortic system for dissections	13,000	105 (Heart valve surgery without cardiac catheterization)	25,000	52.0	Yes	Not available
		110 (Major interventions on the cardiovascular system with complications)	14,200	91.5		
		111 (Major interventions on the cardiovascular system without complications)	10,500	123.1		
4. Tendyne (Abbott Cardiovascular, Plymouth, MN): transcatheter mitral valve replacement	28,000	104 (Heart valve interventions and other major cardiothoracic interventions with cardiac catheterism)	24,115	116.1	Yes	Not available
5. Cardioband (Edwards Lifesciences, Irvine, CA): tricuspid valve reconstruction system	22,000	104 (Heart valve interventions and other major cardiothoracic interventions with cardiac catheterism)	22,115	99.5	Yes	Not available
6. Pascal Mitrale Ace (Edwards Lifesciences, Irvine, CA): mitral valve transcatheter repair system	22,000	104 (Heart valve interventions and other major cardiothoracic interventions with cardiac catheterism)	24,115	91.2	Yes	The cost-effectiveness ratio is favorable considering a Pascal price of 20,000 euros [5]
7. Pascal Tricuspide Ace (Edwards Lifesciences, Irvine, CA): tricuspid valve transcatheter repair system	22,000	104 (Heart valve interventions and other major cardiothoracic interventions with cardiac catheterism)	24,115	91.2	Yes	The cost-effectiveness ratio is favorable considering a Pascal price of 20,000 euros [5]
8. Pascal (Edwards Lifesciences, Irvine, CA): mitral valve transcatheter repair system	20,000	104 (Heart valve interventions and other major cardiothoracic interventions with cardiac catheterism)	22,115	90.4%	Yes	The cost-effectiveness ratio is favorable considering a Pascal price of 20,000 euros [5]
9. Braxon (DECOmed, Marcon, Italy): acellular dermal matrix breast reconstruction	2,939	258 (Total mastectomy for malignant neoplasms without complications)	3,341	88.0	No	Not available
10. Megasystem (Waldemar Link GmbH, Hamburg, Germany): modular shoulder prosthesis	7,000	491 (Interventions on major joints and upper limb reimplant)	8,822	79.3	No	Not available
11. QuiremScout (Quirem Medical B.V., Deventer, The Netherlands): bead diagnostic device	3,000	203 (Malignant neoplasms of the hepatobiliary system or pancreas)	4,208	71.3	No	Not available
12. Micra AV Model MC1AVR1 (Medtronic Europe, Tolochenaz, Switzerland): leadless ventricular pacemaker	8,500	110 (Major interventions on the cardiovascular system with complications)	14,208	59.8	Yes	Not available
13. Cardia Ultrasept Dia (Cardia Inc., Eagan, MN): atrial septal defect closure device	3,750	518 (Percutaneous interventions on the cardiovascular system without stent insertion into the coronary artery without myocardial infarction)	9,881	38.1	Yes	Not available
14. EkoSonic (EKOS Corporation, Bothell, WA): catheter-directed thrombolysis	3,000	075 (Major interventions on the chest)	8,737	34.3	Yes	Not available
15. Impella RP (Abiomed, Danvers, MA): percutaneous ventricular assist device	18,000	525 (Implantation of other cardiac assistance systems)	53,272	33.8	Yes	Not available
16. IntellaNav StablePoint (Boston Scientific, Marlborough, MA): ablation catheter incorporating local impedance data	2,200 to 2,600	555 (Percutaneous interventions on the cardiovascular system with major cardiovascular diagnosis)	9,283	From 23.7 to 28.0	No	Not available
17. Konar VSD Occluder (LifeTech, Petaling Jaya, Malaysia): transcatheter closure of ventricular septal defect	4,000	108 (Other cardiothoracic interventions)	18,389	21.7	Yes	Not available
18. Protek Duo (LivaNova, London, UK): right ventricular assist device	5,350	541 (Extracorporeal oxygenation or tracheostomy with mechanical ventilation of 96 hours or main diagnosis not related to face mouth neck with major surgical intervention)	45,689	11.7	No	Not available
19. His Bundle Kit 3D (BIOTRONIK, Berlin, Germany): introduction system for implantation of leads in sites specifications and lead	500	551 (Permanent cardiac pacemaker implantation with major cardiovascular diagnosis or automatic defibrillator or pulse generator)	9,384	5.3	No	Not available
		552 (Other permanent cardiac pacemaker implants without a major cardiovascular diagnosis)	4,756	10.5	No	Not available
20. Avalus (Medtronic Europe, Tolochenaz, Switzerland): stented bovine pericardial aortic bioprosthesis	2,200	104 (Heart valve interventions and other major cardiothoracic interventions with cardiac catheterism)	23,441	9.4	Yes	Not available
		105 (Heart valve surgery without cardiac catheterization)	19,462	11.3	Yes	
21. BioFreedom (Biosensors International Ltd, Singapore): polymer-free drug-coated stents	515	558 (Percutaneous intervention on the cardiovascular system with a drug-eluting stent without a major cardiovascular diagnosis)	6,434	8.0	Yes	Not available
22. TriGUARD 3 (Keystone Heart, Ltd., Tampa, FL): cerebral protection device	1,950	104 (Heart valve interventions and other major cardiothoracic interventions with cardiac catheterism)	24,115	8.1	Yes	Not available
23. Destino Twist (OSCOR Inc., Palm Harbor, FL): steerable sheath	800	110 (Major interventions on the cardiovascular system with complications)	14,208	5.6	No	Not available
24. Hyalobarrier gel (Nordic Pharma GmbH, Zürich, Switzerland): auto-crosslinked hyaluronan gel for adhesion prevention in laparoscopy and hysteroscopy	145	359 (Interventions on uterus not for malignant neoplasms without complications)	3,027	4.8	No	Not available
		365 (Other interventions on the female reproductive system)	3,059	4.7		
		151 (Lysis of peritoneal adhesions without complications)	4,509	3.2		

Besides Neovasc Reducer, three other devices, i.e., PuraStat (3-D Matrix, Ltd., Tokyo, Japan), Ascyrus Medical Dissection Stent (AMDS, CryoLife, Inc., Kennesaw, GA) when used according to DRG 111, and Tendyne (Abbott Cardiovascular, Plymouth, MN), were found to be priced more than the reimbursement tariff (i.e., ratio > 100%). Ratios between 50% and 100% were found in about half of the devices. Information on cost-effectiveness was not available for most devices. This was mainly due to the insufficient clinical evidence available, which is typical of devices, particularly those in the first phases of marketing. Unfortunately, this lack of information on cost-effectiveness did not allow us to carry out a pharmacoeconomic comparison of most new devices with those already available in our hospitals. This unavailability of information equally affected both the clinical side and the economic one. Finally, from our preliminary assessment on the presence of a post-discharge impact, 15 devices out of 24 (62%) were found to determine a substantial impact after discharge, while the remaining nine (38%) did not.

## Discussion

In the first place, evaluating the post-discharge impact of the 24 devices included in Table 1 was an original finding of our study because, as confirmed in the Appendix, this point has not been addressed in the literature previously. As regards the subgroup of nine devices with no impact after discharge, their clinical-economic impact was restricted to the patient’s in-hospital stay and therefore implied no particular complexity; in fact, when the costs and benefits of a device do not extend beyond the patient's discharge, the presence of a ratio > 100% reliably suggests that either the device price needs to be reduced or the tariff needs to be increased; the analysis in these cases is straightforward irrespective of which decision is needed.

On the other hand, in cases where the device extends its impact beyond the patient’s hospital stay, the decision of either reducing the price or increasing the tariff becomes complex. This is because a large number of factors are involved in both the hospital stay and the post-discharge phase. Among these factors, the clinical benefits achieved by the patient after discharge and the long-term savings resulting from the consequent reduction in healthcare costs are particularly difficult to be assessed. Hence, a full clinical-economic assessment would be needed in these cases, but the analysis is always complex. For example, this scenario applies to Neovasc Reducer, AMDS, and Tendyne, whose ratios between price and tariff are higher than 100%. To a lesser extent, this also applies to Cardioband, Pascal Mitrale Ace, Pascal Tricuspide Ace, Pascal, and Micra, whose price-to-tariff ratio is between 50% and 100%.

In summary, adequate governance of MDs can be achieved when an insufficient DRG is combined with the absence of a post-discharge clinical-economic impact. On the other hand, the analysis is complex when an insufficient DRG is combined with the presence of a post-discharge clinical-economic impact.

When the price/DRG ratio is high and a full economic analysis is unavailable, selecting the most appropriate corrective interventions is difficult; in particular, it is difficult to estimate which monetary increase would be needed in the DRG or which reduction in the device price. This suggests that a sound governance of costs and benefits in the field of high-technology devices is not presently achievable unless the two following points are substantially improved: (1) the quality of patient-related information both in hospital stay and in the post-discharge phase; and (2) the human resources allocated to HTA at public institutions with the purpose of managing and interpreting information on costs and benefits.

Our literature search also identified the previously mentioned paper [1] published by our group in 2020. In comparing the present analysis with that published in 2020, one substantial difference emerges because the ratio price/DRG was calculated for a small number of individual devices in the present analysis whereas, in our previous analysis, this calculation was applied to an entire hospital or an entire region. The implications raised by these two types of analysis are different, but both estimates of this ratio can be useful because, in this way, the same issue is examined from two different perspectives: the one focused on a single treatment or procedure (according to which decisions can be made on device procurement) and the one focused on the hospital or region (according to which decisions can be made about the governance of the healthcare system).

In the light of our findings, an adequate quantification of the main clinical and economic parameters related to the in-hospital use of MDs seems to be unlikely within the present organization of hospitals and the current level of patient traceability. In particular, an improvement is needed in the collection of patients’ in-hospital information as well as in the out-of-hospital monitoring of patient-related events. Until these improvements do not take place, it is difficult to intervene on DRGs owing to this lack of critical information. Well-known delays in the update process of Italian DRGs have also contributed to this negative scenario.

On the other hand, it is also difficult to intervene on device prices, again owing to the lack of critical information. In theory, the price should be proportionate to the extent of clinical benefit, but since the data on the efficacy and safety of MDs are limited, in most cases, one cannot establish which prices are cost-effective and which are not.

Our work has documented a small part of this overall problem since our analysis examined only a small number of devices (i.e., class III and active implantable devices) proposed for inclusion as new products in the regional formulary. To assess the real economic impact of MDs in our NHS and highlight the potential discrepancies between price versus reimbursement tariffs, analyses like ours should hopefully be extended to include the large number of high-cost devices regularly purchased through regional tenders. Potential candidates for these further analyses include implantable defibrillators for which the relationship between price and reimbursement is between 42% and 90%. Likewise, percutaneous aortic valves are another critical device class whose price represents approximately 85% of the DRG.

As regards the limitations of the present work, while conducting our analysis was straightforward owing to its descriptive nature, its main weakness depends on the lack of some essential information that would be required to carry out a cost-effectiveness assessment. The main problem in the Italian national health system (and in the Tuscany region as well) consists in the poor quality of computerized medical records, especially within hospitals. In particular, no efficient systems are available that allow for an exchange of patients' medical records across different hospitals. Instead, the availability of a detailed patient's history is a key factor to adequately assess costs and benefits, especially in chronic diseases that require a long-term follow-up.

In this overall context, the French experience of MD governance, developed over the past few years, is particularly interesting [6]. In France, institutions that manage devices for in-hospital use have in fact undertaken some original pathways of governance. One of these is based on a regularly updated list of MDs, especially the most expensive and innovative ones, that are funded separately from DRG tariffs. In more detail, while most MDs are managed in France according to the “traditional” reimbursement rule based on DRG (“intra-DRG list”), a number of devices are managed separately from DRGs and, in more detail, are included in the so-called “additional list.” These devices are in fact reimbursed outside the DRG based on a separate funding pathway [6].

Finally, it should be kept in mind that while the combination of an insufficient DRG with the presence of a post-discharge clinical-economic impact is an important negative factor in terms of governance, other factors are involved too. Among these, one seems to be particularly important: the combination of an insufficient DRG with the presence of post-discharge clinical benefits encourages, albeit unintentionally, the inter-regional mobility from less developed regions toward more developed regions (where the degree of development is intended in terms of quality of hospital health care). In fact, the technologically “backward” regions, which have not equipped themselves to promptly implement innovations at their own facilities, are inappropriately rewarded if they promote regional mobility of specific patients so that they reimburse - at low DRG tariffs - patients treated with innovative services that other more advanced regions have implemented and can provide to these patients. At the same time, these less advanced regions can derive an undeserved economic advantage from the reduction in healthcare costs incurred by patients in their region of residence, thanks to the successful treatment received in another region [7].

A final point deserves to be mentioned. In the Tuscany region, the yearly expenditure for MDs is, more or less, similar to that of pharmaceutical products. The expenditure for MDs is mainly restricted to hospitals in a context where the upwards trend in this expenditure is growing rapidly. In contrast, a large part of the expenditures for pharmaceuticals takes place in the community where the economic trend is stable. This trend observed in the Tuscany region for both devices and pharmaceuticals is essentially the same as in the other Italian regions.

## Conclusions

In the field of MDs, the current scenario in terms of governance is likely to improve over the next years. Some Italian regions such as Tuscany have implemented new HTA activities specifically focused on MDs. One such example is represented by the analysis presented herein. Although the scientific literature about MDs remains scarce in Italy, the presence of these new activities will hopefully yield positive effects on the regional governance of these products and, consequently, also in terms of scientific impact.

## Figures and Tables

**Figure 2:**
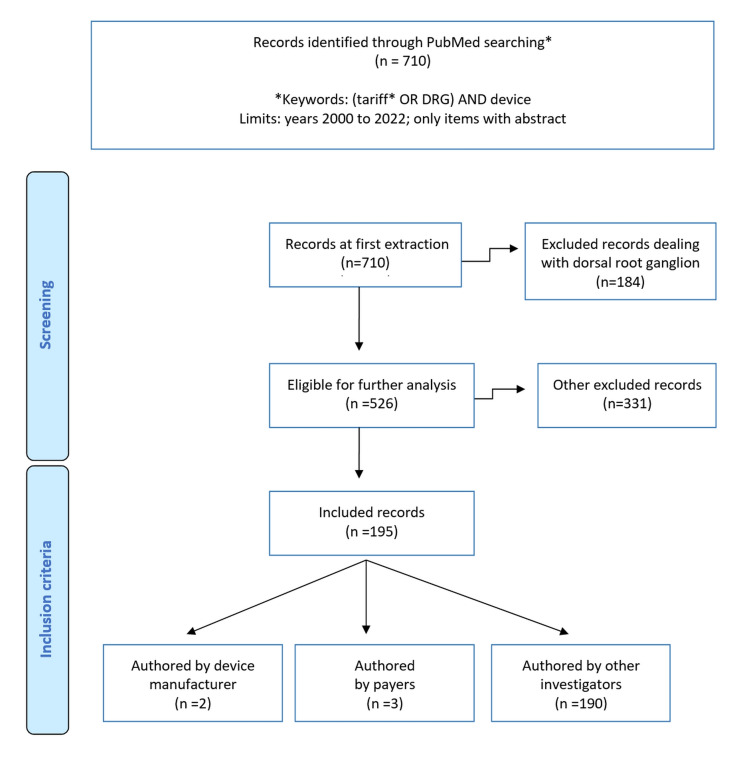
PRISMA flow diagram of our PubMed search. PRISMA, Preferred Reporting Items for Systematic Reviews and Meta-Analyses.

## Appendices

This appendix presents a PubMed search of articles focused on the relationship between device price and DRG tariff. Figure 2 shows the PRISMA (Preferred Reporting Items for Systematic Reviews and Meta-Analyses) flow chart of this search (and related keywords). A total of 710 citations were selected at first extraction; then, a subset of “pertinent” articles (n = 195) was identified by checking abstracts individually and selecting articles that dealt with one or more devices as well as their DRG tariffs. Investigators who authored these articles were in a large proportion clinicians who worked in a hospital setting (n = 190; 97%). A small number of these articles (n = 2; 1.5%) were authored by the institution responsible for the payment of the procedure. Only two studies (1%) were authored by the manufacturer of the device. The overall picture that emerges from this PubMed search is characterized by an overall small number of studies on these topics, given that the entire world literature was examined. On the other hand, the group of 195 “pertinent” articles showed a clear fragmentation of results because their focus was generally limited to a single device or procedure, and no systematic review was found that evaluated different devices or procedures in terms of price and tariffs. Further details on the 710 articles included in this analysis are available as a preprint in reference [8].

## References

[1] Messori A, Trippoli S, Marinai C (2020). The role of medical devices in influencing in-hospital sustainability: an analysis of expenditure in 2019 vs DRG reimbursement according to major medical specialties in a region of middle Italy. Expert Rev Med Devices.

[2] (2016). Determinazione delle tariffe regionali per il pagamento delle prestazioni di ricovero ospedaliero per acuti in vigore dal 10 ottobre 2016. Bollettino Ufficiale della Regione Toscana n.40 del 5.10.2016.

[3] (2022). Regione Toscana. Prodotti HTA - Schede HTA. https://www.regione.toscana.it/-/prodotti-hta.

[4] Gallone G, Armeni P, Verheye S (2020). Cost-effectiveness of the coronary sinus reducer and its impact on the healthcare burden of refractory angina patients. Eur Heart J Qual Care Clin Outcomes.

[5] Shore J, Russell J, Frankenstein L, Candolfi P, Green M (2020). An analysis of the cost-effectiveness of transcatheter mitral valve repair for people with secondary mitral valve regurgitation in the UK. J Med Econ.

[6] (2022). Haute Autoritè De Santè. Pathway of medical devices in France. Practical guide. November.

[7] (2022). Interrogazione e risposta scritta del Governo - Sulla TAVI (transcatheter aortic valve implantation). https://portale.fnomceo.it/interrogazione-e-risposta-scritta-del-governo-sulla-tavi-transcatheter-aortic-valve-implantation/.

[8] Messori A, Trippoli S. (2022). List of 710 citations selected from PubMed based on the keywords “tariff”, “DRG”, and “device” (time period: January 2020 to February 2022). Open Science Framework, access 4 March 2022..

